# Dividing Attention Between Tasks: Testing Whether Explicit Payoff Functions Elicit Optimal Dual‐Task Performance

**DOI:** 10.1111/cogs.12513

**Published:** 2017-06-27

**Authors:** George D. Farmer, Christian P. Janssen, Anh T. Nguyen, Duncan P. Brumby

**Affiliations:** ^1^ UCL Interaction Centre University College London; ^2^ Division of Neuroscience & Experimental Psychology University of Manchester; ^3^ Experimental Psychology & Helmholtz Institute Utrecht University; ^4^ Department of Psychological Sciences University of Missouri

**Keywords:** Multitasking, Rational behavior, Optimization, Cognitive control, Task interleaving, Time allocation

## Abstract

We test people's ability to optimize performance across two concurrent tasks. Participants performed a number entry task while controlling a randomly moving cursor with a joystick. Participants received explicit feedback on their performance on these tasks in the form of a single combined score. This payoff function was varied between conditions to change the value of one task relative to the other. We found that participants adapted their strategy for interleaving the two tasks, by varying how long they spent on one task before switching to the other, in order to achieve the near maximum payoff available in each condition. In a second experiment, we show that this behavior is learned quickly (within 2–3 min over several discrete trials) and remained stable for as long as the payoff function did not change. The results of this work show that people are adaptive and flexible in how they prioritize and allocate attention in a dual‐task setting. However, it also demonstrates some of the limits regarding people's ability to optimize payoff functions.

## 1 Introduction

With the growing ubiquity of mobile technology, people regularly interleave attention between concurrent tasks. Human multitasking occurs in a variety of task domains and is investigated across many disciplines (e.g., see special issue; Janssen, Gould, Li, Brumby, & Cox, 2015). Everyday multitasking can have implications for safety, such as when a driver uses a phone while driving (Dingus et al., 2016), as well as implications for productivity, such as when an office‐worker is having a conversation with a colleague while working on a spreadsheet (González & Mark, 2004). Fundamentally when people are faced with two or more tasks, they are faced with a scheduling problem: deciding how much time to spend on one task before shifting attention to the next task (Moray, Dessouky, Kijowski, & Adapathya, 1991). Given the prevalence of multitasking, it is important to understand how well people deal with the problem of allocating their attention between tasks.

In this paper, we investigate how priorities, as formalized through an explicit payoff function, affect how people choose to allocate their attention when multitasking. We report the results of an experiment in which participants had to perform two independent tasks. Each of these tasks gave a reward based on how well the task was performed. Critically, participants could not perform both tasks at the same time, and so had to decide how to divide effort between them. We examine whether participants were able to interleave tasks in such a way as to earn the maximum amount of overall reward that was achievable for them. In other words, we consider whether participants were able to settle on the optimal dual‐task strategy. This approach allows us to understand how good people are at dividing attention between multiple concurrent tasks, and it provides insights into the cognitive constraints that might otherwise limit or prevent people from achieving optimal performance in a demanding multitasking environment.

Existing multitasking research has highlighted several important factors that influence how people interleave tasks. One such factor is the characteristics of the task itself. People may avoid combining tasks of similar modalities in order to prevent interference between them (Salvucci & Taatgen, 2008, 2011; Wickens, 2002, 2008). A task can have “natural breakpoints” (Janssen, Brumby, & Garnett, 2012), where interleaving is particularly beneficial, for example, because workload is low at those points (Bailey & Iqbal, 2008; Salvucci & Bogunovich, 2010) or because these natural breakpoints incur shorter resumption costs after an interruption (Altmann & Trafton, 2002; Borst, Taatgen, & van Rijn, 2010). Other researchers debate people's ability to multitask optimally (e.g., Nijboer, Taatgen, Brands, Borst, & van Rijn, 2013; Ophir, Nass, & Wagner, 2009; Stoet, O'Connor, Conner, & Laws, 2013; Watson & Strayer, 2010), focusing on the interplay between cognitive characteristics and task performance (e.g., “do people perform worse when they are faced with two challenging tasks?”) and on performance decrements in multitask compared to single‐task settings.

When multitasking, people often have to choose how tasks should be prioritized relative to one another (e.g., Gopher, 1993; Gopher, Brickner, & Navon, 1982). For example, in our previous work examining in‐car multitasking (Brumby, Salvucci, & Howes, 2009; Janssen & Brumby, 2010; Janssen et al., 2012), we found that varying instructions for how a phone dialing task should be prioritized relative to the driving task affected how participants chose to interleave these two tasks. Participants who were instructed to prioritize safer driving achieved this by interacting with the phone in shorter bursts of activity (i.e., by dialing fewer digits at a time). In contrast, participants who were instructed to prioritize the dialing tasks achieved this by interacting with the phone in longer bursts, resulting in poorer driving performance. A limitation of this prior research on the effect of task priorities on multitasking strategy is that instructions like “prioritize safer driving” represent a subjective criterion of performance: It is unclear to both the participant and the researcher just how much one task should be prioritized relative to the other.

Some researchers have attempted to overcome this problem by giving participants clearer instructions as to how tasks should be prioritized. For example, participants might be told: “you should give Task‐A 80% of your attention and Task‐B 20% of your attention” (e.g., Navon & Gopher, 1979; Norman & Bobrow, 1975). While such instructions tell participants how they should aim to divide their attention between tasks, there are many possible ways in which this outcome might be achieved, and there are consequences of this strategy variation for performance. As demonstrated by Brumby and colleagues (Brumby et al., 2009; Janssen & Brumby, 2010; Janssen et al., 2012), dual‐task performance often depends on low‐level decisions about how tasks are interleaved (i.e., the time spent on a task before switching to another task).

In this study, we investigate human multitasking behavior by focusing on how people decide to interleave two concurrent tasks. We gave participants an explicit reward based on how well each task was performed; this reward is represented as points earned on a given trial. Critically, each task makes an independent contribution to the reward total, which allows for the relative value of one task to the other to be systematically varied. With this setup, we investigate how changing task weighting affects the dual‐task interleaving strategy that participants choose to adopt. A particular benefit of this approach is that it allows us to formally assess how “good” the strategy adopted is: Do people select strategies that earn them the most points available (i.e., the optimal multitasking strategy)?

### 1.1 Testing strategy flexibility through payoff manipulation

Payoff functions can be used to test people's ability to optimize performance on tasks that have competing dimensions like speed and accuracy. These functions translate performance on each separate dimension of a task into a single unit of reward, which can then be fed back to the participant in terms of the number of points accrued over the course of an experiment.

In a dual‐task setting, a payoff function can communicate performance trade‐offs between tasks to the participant, indicating how one task should be prioritized relative to the other; whereas a verbal instruction might allow for multiple subjective interpretations of what constitutes optimal performance, an explicit payoff score does not. A payoff function means that reward can be accrued (either explicitly or implicitly) and therefore can give participants a sense of progress and influence how they interleave tasks (Duggan, Johnson, & Sørli, 2013; Payne, Duggan, & Neth, 2007). This can be used to motivate the participant to try to achieve the maximum score (e.g., Hornof, Zhang, & Halverson, 2010; Schumacher et al., 1999; Wang, Proctor, & Pick, 2007; Zhang & Hornof, 2014) and help them to learn better ways of dividing attention between tasks (Erev & Gopher, 1999).

When combined with computational cognitive models, payoff functions can be used to explore how changes to the function (i.e., the relative weight of one task to another) affect overall performance, and whether people can apply strategies that maximize reward (Howes, Lewis, & Vera, 2009; Janssen, 2012; Janssen & Brumby, 2015; Janssen, Brumby, Dowell, Chater, & Howes, 2011; Lewis, Howes, & Singh, 2014; Payne & Howes, 2013). There is good evidence that people will seek to maximize reward in tasks where the optimal strategies are tractable and where feedback is available (see special issue, Howes, Lewis, & Singh, 2014; and work on decision‐making in which performance is measured in terms of efficiency, e.g., Jarvstad, Hahn, Rushton, & Warren, 2013).

The analysis of optimality in human performance can be used to model the interaction between an agent's goal and her cognitive constraints. Previous studies have shown that people can optimize payoff functions to maximize reward in simple dual‐task scenarios, such as the Psychological Refractory Period task (Howes et al., 2009) and in discretionary multitasking scenarios (Janssen, 2012; Janssen & Brumby, 2015; Janssen et al., 2011; Zhang & Hornof, 2014). In these studies, participants adapted performance to different levels of difficulty of the task, given the payoff function at hand. However, these studies have often used a stable and consistent payoff function, rather than varying it and seeing how people respond and whether they adapt their behavior according to changes to the function. This study is aimed at testing the claim that people can optimize payoff (reward) functions in dual‐task settings. Specifically, the experiments and associated models were designed to expose participants to a variety of different payoff functions for the same dual‐task scenarios. Across conditions, participants had to be flexible and adapt their strategy in order to achieve the optimal payoff.

For the experiments reported here, we use a modified version of the task environment that was developed by Janssen and colleagues (Janssen, 2012; Janssen & Brumby, 2015; Janssen et al., 2011). In this dual‐task setup, participants have to type in a string of digits while also making sure that they keep a randomly moving cursor within a target area. Janssen and Brumby (2015) demonstrated that people change their task interleaving strategy based on the weight given to each task in the payoff function. Specifically, when the payoff functions weighted the reward of one of the tasks (e.g., typing in digits), participants paid longer visits to that task compared to the other, less weighted task (e.g., tracking a cursor). However, this study presented several limitations. First, characteristics of the participants, such as their typing speed, strongly affected their performance, which made it difficult to compare strategies between participants. In other words, the optimal dual‐task interleaving strategy for a given condition was dependent on an individual's performance on the component tasks (e.g., faster typists would have optimal visit durations different from slower typists). Accounting for such individual differences is challenging. Second, within each participant group, we tested the effect of different task characteristics while keeping the payoff function constant. Some participants then transferred previously successful strategies to this new setting, even though the strategies were now suboptimal.

Here, we seek to redress issues with Janssen and Brumby's (2015) study by testing how different payoff functions affect the ability to optimize task interleaving. To do this, we examine each participant's capacity to perform optimally while taking into account his or her individual cognitive constraints, by modeling the full range of task interleaving strategies (how long to spend on each task) available to each participant given each participant's individual performance profile on the component tasks. In addition, we use a within‐subjects design in Experiment 1 to test whether people can adapt to changes in payoff function (rather than the between‐subjects design used by Janssen & Brumby, 2015). The benefit of this approach is that contrasts between conditions can be identified even when there are individual differences in skill (more specifically, in Janssen & Brumby, 2015, there were some differences in skill between groups, which counteracted some of the payoff manipulations). However, it does come at the risk of strategy transfer between conditions.

In the current experiments, we used three different payoff regimes to systematically vary the importance of one task relative to the other. Specifically, we changed the penalty that was applied for failing to attend to the tracking task (details below). We were interested in whether participants were sensitive to these changes to the payoff function and whether it would influence the time participants chose to spend on one task before switching back to the other.

Based on the observation that people have maximized rewards in previous multitasking studies (e.g., Howes et al., 2009; Janssen, 2012; Janssen et al., 2011; Zhang & Hornof, 2014) and in other settings (e.g., Gray, Sims, Fu, & Schoelles, 2006; Lewis, Shvartsman, & Singh, 2013; Lewis et al., 2014; Myers, Lewis, & Howes, 2013), we again predict that participants in our study should be able to adapt their behavior to maximize payoff. More specifically, we hypothesize that participants will adapt their strategy when a change in the payoff function makes a different strategy optimal. We also hypothesize that participants will apply strategies that achieve optimal performance as predicted by a cognitive model for different experimental conditions, which vary both in terms of the payoff function and the difficulty of the tasks being performed.

### 1.2 Reward manipulations and their relationship with everyday life

A key feature of the current experiments is the manipulation of payoff function (or the reward given to one task relative to the other). In everyday life, rewards can take many forms, making it challenging to capture all forms in a single experiment. Three critical aspects that characterize rewards are the timing of the reward, the objective function (i.e., what is rewarded?), and the magnitude of the reward (Janssen & Gray, 2012). In this study, we keep the timing of rewards aligned with a specific event in the task, that is, after participants switch from typing to tracking. We also keep the objective function comparable: All rewards relate to the duration of typing (i.e., how many digits are typed?) and to the accuracy of tracking (i.e., did the cursor leave the target area?)

We therefore chose to manipulate payoff by varying the severity of the penalty imposed for letting the cursor move outside the target area in the tracking task. This setup reflects situations where inattention to one task (tracking) can result in losses on the task on which you previously gained (typing). We manipulate the cost of distraction in tracking in three ways and illustrate the motivation for each function with an analogy to driver distraction.

First, sometimes inattention can have severe consequences. For example, in a driving context you might end up in an accident. We capture this situation in the *lose 500* payoff condition: If the cursor moves outside of the target area while you type, you are given a severe penalty of 500 points, most likely meaning that it will be difficult to obtain a positive total.

Second, in other situations, inattention might result in no progress, but also no actual damage. For example, while driving in an unfamiliar area, you might not realize that you have taken the wrong turning and substantially delay your arrival at your destination. We capture this with the *lose‐all* condition: All the accumulated points are lost, but the total cannot go negative.

Third, sometimes the losses you incur are relatively minor. If you fail to pay attention when driving a very familiar route, you might miss the turning for the quickest route home, but you can easily take the next turning without adding much time to your journey. The experiment captures this situation in the *lose‐half* condition, where half the accumulated points are lost if the cursor moved outside of the target area.

To summarize, previous work has made claims about humans’ ability to optimize task interleaving in various situations, including dual‐task studies, provided that a clear payoff function is given. The aim of the current work is to test this general claim further, by testing the capabilities and limitations of people's ability to divide their time efficiently between two tasks. In particular, if participants change their strategy in response to a change in the payoff function, can they find and select the best strategy for maximizing reward under the current payoff regime?

## 2 Experiment 1

### 2.1 Methods

#### 2.1.1 Participants

Twenty students (seven female) from University College London participated on a voluntary basis. Participants were between 22 and 37 years of age (*M *=* *27 years). Participants were not paid for their time, but an incentive of a £10 voucher was offered for the participant who achieved the highest score in the study.

#### 2.1.2 Materials

**Figure 1:**
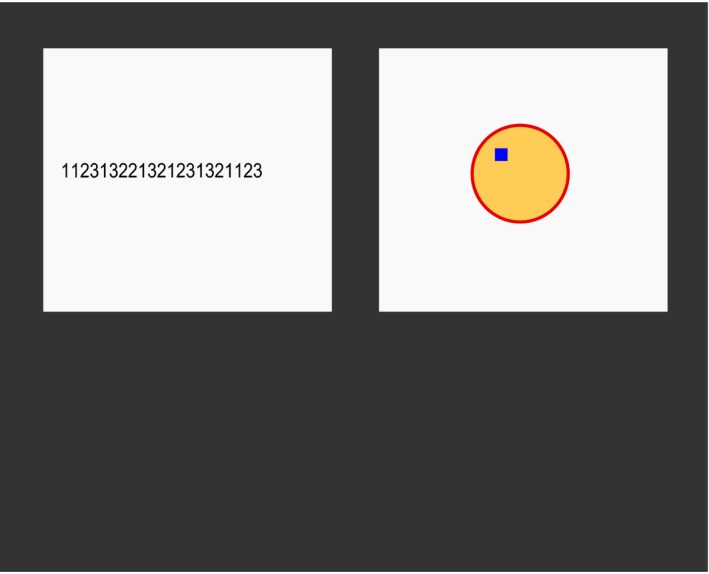
Position of the two task windows in the interface. Only one of the task windows was visible at a time. Participants could switch between windows by pressing or releasing a button on the joystick (see text for details).

A dual‐task setup similar to that of Janssen et al. (2011) was used, in which participants completed a discrete typing task while monitoring a continuous tracking task (see Fig. 1). Tasks were displayed on a 17‐inch monitor at a resolution of 1,024 by 1,280 pixels, with each task being presented within a 450 × 450 pixel area. The typing task was presented on the left side of the monitor, and participants entered digits with their left hand using a numeric keypad. The tracking task was presented on the right side of the monitor and participants controlled the task with their right hand using a Logitech Extreme 3D Pro joystick. There was a horizontal separation of 127 pixels between the two tasks. At any moment in time, only one of the tasks was visible on the monitor. By default, the typing task was visible and the tracking task was covered by a gray square. Holding down the trigger of the joystick revealed the tracking task and covered the typing task. Releasing the trigger covered the tracking task and made the typing task visible again. Each task could only be controlled when it was visible.

#### 2.1.3 Typing task

In the typing task, participants were required to enter digits using a numeric keypad. A continuous list of to‐be‐entered digits was generated from the numbers 1–3 drawn in a random order with the constraint that no digit was repeated more than three times in a row. At any one moment, 27 digits were visible on the typing task display. As a participant typed, the left‐most digit in the list would disappear, all digits would then move one to the left, and a new digit would appear in the right‐most position. Each correctly entered digit earned a participant 10 points. The display remained unchanged if an incorrect digit was entered and a penalty of minus five points was applied.

#### 2.1.4 Tracking task

For the tracking task, participants were required to keep a square cursor inside a circular target area. The cursor was 10 × 10 pixels and the target area had a radius of 120 pixels. The movement of the cursor was updated every 23 ms. Values were sampled from a Gaussian distribution to determine the size of the cursor's movement, and the parameters on this distribution were varied between conditions in such a way to make the cursor move about at different speeds. We used two types of cursor noise: high noise (5 pixels standard deviation) and low noise (3 pixels standard deviation). Holding down the trigger of the joystick allowed participants to see the tracking task and move the cursor around with the joystick if they so wished. Releasing the trigger covered up the tracking task again. While participants were typing, there was no feedback available concerning the status of the tracking task. The only way to determine the position of the cursor, and whether it was still in target area, was by switching to the tracking task. Due to the nature of the random drift function, the cursor could move outside the target area, and move back in again, while the participants were typing. When participants switched to the tracking task, they would see a red cursor if it had left the target area at any point during the previous typing visit. A red cursor was reset to blue by dragging it toward the center of the target area.

#### 2.1.5 Dual‐task

Using this dual‐task setup, participants completed a series of trials, each lasting 120 s. During each trial, the main decision facing the participant was to judge how long she should leave the tracking task unattended while entering digits. Participants received feedback on their performance in terms of the number of points achieved after each visit to the typing task. The payoff function rewarded participants for each digit that was correctly entered during the visit, and it penalized them for entering digits incorrectly. An additional penalty was also applied if the cursor drifted outside the target area during the visit. The precise nature of the payoff function was varied between conditions. Feedback was displayed above the tracking task and remained visible while participants were tracking. Participants could therefore evaluate their performance after each visit to the typing task. Cumulative feedback was also given at the end of each trial.

#### 2.1.6 Design

A 3 × 2 (payoff function × cursor noise) within‐subjects design was used. In each of the payoff function levels, points were deducted if the cursor drifted outside the target area in the tracking task. The severity of this tracking penalty was varied such that participants either lost all the points gained for that visit (lose‐all condition), half of the points gained for that visit (lose‐half condition), or incurred a fixed penalty of 500 points (lose 500 condition). Because participants typically only typed for a few seconds, a 500‐point penalty could make the total score negative, making the lose 500 condition the most severe of the three conditions.

The two levels of cursor noise factor, low noise and high noise, influenced how much the cursor drifted on each update. Each cursor update was sampled from a Gaussian function with a mean of zero and a standard deviation of 3 pixels (low noise condition) or 5 pixels (high noise condition).

The dependent variable of interest was the mean visit duration to the typing task. This measure captures the trade‐off that participants had to make between gaining points (by typing more digits) and losing points (by incurring the tracking penalty). We also collected data on how far participants allowed the cursor to drift, the amount of time they spent on the tracking task, and their typing speeds.

#### 2.1.7 Procedure

Upon arrival, participants received instructions on the dual‐task setup. Crucially, they were briefed that they could gain points in dual‐task trials through fast and accurate typing, and that they would lose points by making typing errors and by letting the cursor drift outside the target area. Participants were not informed of the exact way in which the gain and penalties were calculated in each condition. They were told that the payoff function changed between blocks of trials.

At the beginning of the experiment, participants were given a chance to practice each of the tasks separately (tracking for two trials of 10 s each and typing for two trials of 20 s each) before being given a chance to practice the tasks together (for two trials of 30 s each). Following the practice session, participants completed six experimental blocks, one for each condition. For each block, participants first completed two single‐task tracking trials of 10 s each so that they could estimate the noise on the cursor movement, and two single‐task typing trials of 20 s each. There were then two dual‐task trials of 120 s each. The first trial allowed participants to become familiar with the dual‐task and payoff function. We report data from only the second of each dual‐task trial from each block (condition). In total, the experiment took about 45 min to complete.

Participants were given a brief break after every other block of trials. The order in which the different payoff conditions were experienced was randomized and counterbalanced across participants. The order of the noise conditions was assigned randomly for the first two blocks, but repeated for each payoff condition.

### 2.2 Results

**Figure 2:**
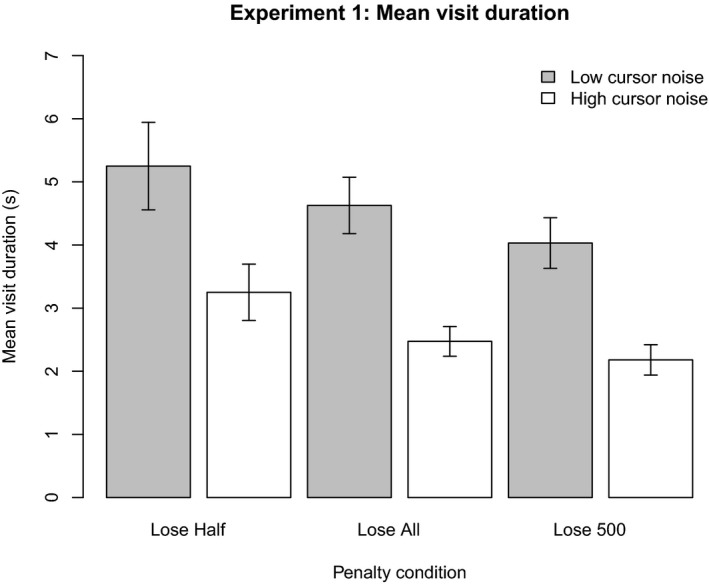
Mean visit duration to the typing task in each condition. Error bars are standard error of the mean.

Fig. 2 shows the mean visit duration to the typing task in each of the six conditions grouped by amount of noise (bar color) and payoff function. The severity of the payoff function's penalty increases from left to right: lose‐half, lose‐all, lose 500. For all statistical analyses, we used a 3 × 2 (payoff function × cursor noise) repeated‐measures analysis of variance (anova), with a significance level of *p *=* *.05.

Participants made shorter visits when the penalty for having the cursor go outside of the target area was more severe. Similar patterns were observed in other measures recorded, such as the distance the cursor was allowed to drift and the number of digits typed (see Supporting Information). Statistical analysis on the dependent variable of mean visit duration to the typing task showed that there was a significant main effect of payoff function, *F*(2*,* 38) = 5.10, *p *=* *.011, $\eta_{p}^{2}=0.21$. A Bonferroni‐corrected post hoc test found that visits were 1.15 s shorter in the lose 500 condition (*M *=* *3.11 s, 95% CI = [2.50, 3.71]), compared to the lose‐half condition (*M *=* *4.25 s, 95% CI = [3.09, 5.41], *p *=* *.018). The visit duration in the lose‐all condition (*M *=* *3.55 s, 95% CI = [2.92, 4.19]) did not differ significantly from the other two conditions (*p > *.2).

It can also be seen in Fig. 2 that participants made shorter visits to the typing task when the tracking task was more demanding. There was a significant main effect of cursor noise on visit duration, *F*(1*,* 19) = 61.12*, p < *.001, $\eta_{p}^{2}=0.76$. Participants spent 2 s more per visit when the cursor noise was low (*M *=* *2.64 s, 95% CI = [2.09, 3.18]) compared to when it was high (*M *=* *4.64 s, 95% CI = [3.70, 5.57]). There was no significant interaction effect, *F*(2, 38)* *= 0.25.

### 2.3 Model of optimal performance

Although the above results demonstrate that participants changed their interleaving strategy with both characteristics of the task and the payoff function, what is not yet clear is how good their performance was. Specifically, did participants apply those dual‐task interleaving strategies that, on average, maximized the payoff on the task? To assess this, we developed a model of optimal performance for our task.

The objective of this type of modeling is to understand whether people adopt the best strategies out of the set of possible strategies available to them. This is an analysis toward the computational level of Marr's types of analysis (c.f. Marr, 1982; Peebles & Cooper, 2015). We assume that when given experience with a payoff function and feedback on their performance, people will act rationally under constraints (Howes et al., 2009; Lewis et al., 2014). In other words, participants will try and hone in on and adopt dual‐task interleaving strategies that achieve the maximum reward available to a participant given his or her individual abilities.

There are two key aspects to the model, the first of which is to identify the invariant aspects of human performance which constrain performance on each task. We outline our assumptions regarding these constraints in the following section. The second aspect of the model is to determine the range of strategies available to participants given their individual constraints. Identifying all the available strategies then allows us to determine whether people settle on the best performing of these strategies.

#### 2.3.1 Assumptions: Participant parameters

We assume that participants spend some time typing with a given speed and accuracy, then they switch to the tracking task and return the cursor to the center of the target area. This process is assumed to iterate until the task time limit is reached (120 s in Experiment 1). Rather than modeling the fine‐level structure of each of these tasks, we approximate each action based on measured behavior. This approach is close to the models developed by Janssen et al. (2011), Janssen (2012), and Janssen and Brumby (2015), and it is similar to the use of approximations in engineering‐focused models, such as those described by Card, Moran, and Newell (1983). Although milliseconds can matter in perception‐action routines (Gray & Boehm‐Davis, 2000), the level of abstraction used here has proven useful before in exploring multitasking behavior (e.g., Brumby et al., 2009; Janssen & Brumby, 2010, 2015; Janssen et al., 2011, 2012).

We identified three parameters measured from each participant's performance on the task. These parameters are the typing accuracy, typing speed, and the amount of time taken to return the cursor to the center of the tracking task. Given these mean parameters, the model explores the payoff that each participant would achieve for every possible duration of visit to the typing task. The typing task visit duration is the key strategic decision that a participant must make while engaged in the task.

During the second dual‐task trial, for each participant, we measured the mean key‐press interval (the group level mean was 0.39 s*, SD *= 0.09 s). We also measured each participant's mean typing accuracy (group level: *M *=* *94%, *SD* = 3%). For the tracking task, we measured each participant's mean time spent away from the typing task while attending to the tracking task (the group level parameters were: *M *=* *1.32 s, *SD *= 0.25 s) and assumed that participants always brought the cursor back to center of the target area at the end of their visit.^1^

For each individual, we calibrated the model parameters to his or her measured performance during the task. There were three key parameters which were taken from participants’ performance during the second of the two dual‐task trials they completed in each condition. In the model, we assume that these parameters are constant, reflecting the abilities of each participant rather than a strategic decision. The key decision that the participants did have to make was how long they should spend on visits to the typing task. This mean visit duration to the typing task is what we refer to as the participant's strategy. On the one hand, a long visit duration will accrue many points but will also increase the likelihood that a penalty will be incurred in the tracking task. On the other hand, a very short visit duration will decrease the likelihood of incurring a tracking penalty but will mean less of the trial duration is spent accruing points because the participant would lose time by constantly switching between the tasks.

#### 2.3.2 Calculating the payoff for different strategies

We designed the dual‐task payoff functions such that participants earned 10 points for every correct key press and lost 5 points for every incorrect key press. In order to determine each participant's expected gain for every possible duration of visit, the model calculated the product of each participant's accuracy rate, their number of digits typed, and the gain (10). From this value, the model subtracts the expected cost of incorrectly typed digits, that is, the product of error rate, digits typed, and error cost (−5). The number of digits typed was calculated as the typing visit duration divided by the participant's key‐press interval.

The expected penalty for a typing visit was calculated as the probability of the cursor's exit for the amount of time spent typing, multiplied by the penalty amount. Note that the penalty amount was an independent variable (payoff function) in our design. The three levels were as follows: loss of all points accrued, loss of half the points accrued, and a fixed penalty of 500 points. The overall payoff for a visit is given by the expected gain plus the expected penalty.

While participants were typing, they had to leave the tracking task unattended. The probability of a participant incurring a tracking penalty was therefore a function of the amount of time they spent typing. The cursor in the tracking task was on a random walk from the center of a circular target area with a radius of 120 pixels. The noise on the cursor movement (either low or high) varied according to the condition.

**Figure 3:**
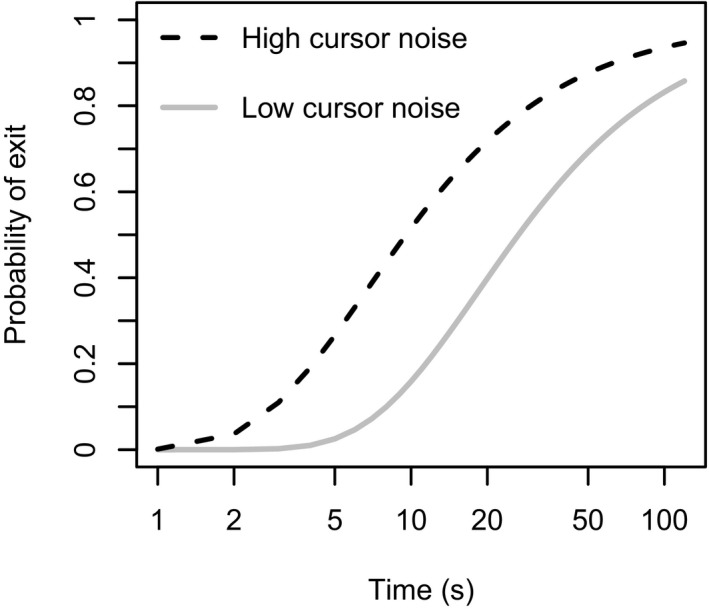
The probability that the randomly moving cursor would exceed the target area for a given typing task visit duration. Note that the horizontal axis uses a logarithmic scale.

Fig. 3 shows the probability of the cursor location exceeding the radius of the target area at a given time. This probability is governed by a cumulative Rayleigh distribution (Walck, 2007) scaled to the duration of the participant's visit and the standard deviation of the cursor movement (dependent on the experimental condition). The probability that the cursor would exceed the target area (i.e., 120 pixels from the origin) is given by:

$$p\left(\text{exit}\right)=\text{exp}\left(-\frac{120^{2}}{2\left(\frac{T}{\lambda }\right)\sigma^{2}}\right)$$

where *T *= typing visit duration, σ = the standard deviation of the random movement, and λ = the interval between cursor position updates (0.023 s). Note that σ was an independent variable (cursor noise) in our design with two levels, either 3 (low noise) or 5 pixels (high noise).

In order to prevent the cursor from exiting the target area, participants had to periodically switch to the tracking task, move the cursor back to the center of the target area, and switch back to the typing task. The mean total duration of these three actions was 1.32 s (*SD *= 0.25). During this time, no gain could be accrued. The time loss incurred by switching to the tracking task, moving the cursor, and switching back to the typing task is represented by the parameter *k*. Since the duration of each trial was 120 s, the number of typing visits is determined as $\frac{120}{T+K}$ where *T* is the mean typing visit duration.

In order to maximize reward over the entire trial, the key strategic decision is to figure out how much time to spend on each visit to the typing task. Typing many digits accrues more gain but incurs greater risk of a penalty. Typing fewer digits reduces the risk of incurring a penalty but results in many switches during which time no gain can be accrued. We derive precise predictions about this trade‐off by having the model evaluate the entire possible strategy space (i.e., all possible variations of visit duration to the typing window before returning attention to the tracking task), from typing a single digit per visit, to spending the entire trial typing and never switching to the tracking task. For each of these strategies we use the model to calculate the payoff that they would achieve.

### 2.4 Model results

In order to determine whether our participants performed *optimally* in selecting their interleaving strategy, we analyzed how optimal agents would have performed. For each participant, we ran the model using his or her individual parameters (typing speed, error rate, and tracking time). As outlined above, we ran the model through the entire strategy space, systematically exploring different alternatives for visit durations to the typing task. For each strategy alternative, we determined how many points would have been earned on average. This analysis allowed us to identify for each participant, the individual optimal visit duration (i.e., the strategy that would earn the most points on average). Note that each individual participant could have a different optimal visit duration, due to variations in typing speed, accuracy, and tracking time. We first consider whether participants could achieve the maximum points as predicted by their optimal model. We then examine how participants’ performance was affected by the shape of the payoff function in each condition.

Table 1 shows, for each condition, the mean score achieved by participants in the study and the predicted mean score of the optimal dual‐task strategies. There was a good level of correspondence between predicted optimal scores and those achieved by participants in each condition, *R* ^2^ = 0.97, *t*(4) = 12.80, *p* < .001. However, despite this seemingly good fit between model and data across conditions, participants consistently achieved fewer points than predicted by the optimal model, *RMSE *= 466 points. A series of *t*‐tests were performed comparing model and data for each condition. To do this, we compared the optimum score predicted for each participant against his or her actual performance in each condition. As can be seen in the table, in all conditions, participants’ performance was significantly worse than the predicted optimum (i.e., all *p*'s < .05).

**Table 1:** Comparison of participants' mean scores and their predicted optimal score

Payoff	Cursor Noise	Participants' Score (*SD*)	Optimal Score (*SD*)	*t*(19)	*p*
Lose‐half	Low	2,041 (425)	2,345 (490)	−4.30	<.001
	High	1,637 (362)	1,986 (423)	−4.85	<.001
Lose‐all	Low	2,055 (509)	2,327 (501)	−4.14	<.001
	High	1,608 (394)	1,944 (514)	−4.41	<.001
Lose 500	Low	1,799 (626)	2,045 (401)	−2.85	.010
	High	323 (872)	1,240 (628)	−3.95	<.001

We next consider the shape of the payoff function for each condition. In other words, how the number of points earned varies depending on the duration of visits to the typing task. This analysis is important because we want to understand how the shape of the payoff function differed between conditions (i.e., in terms of the position of the peak of the payoff function and also the precise shape of the payoff function).

**Figure 4:**
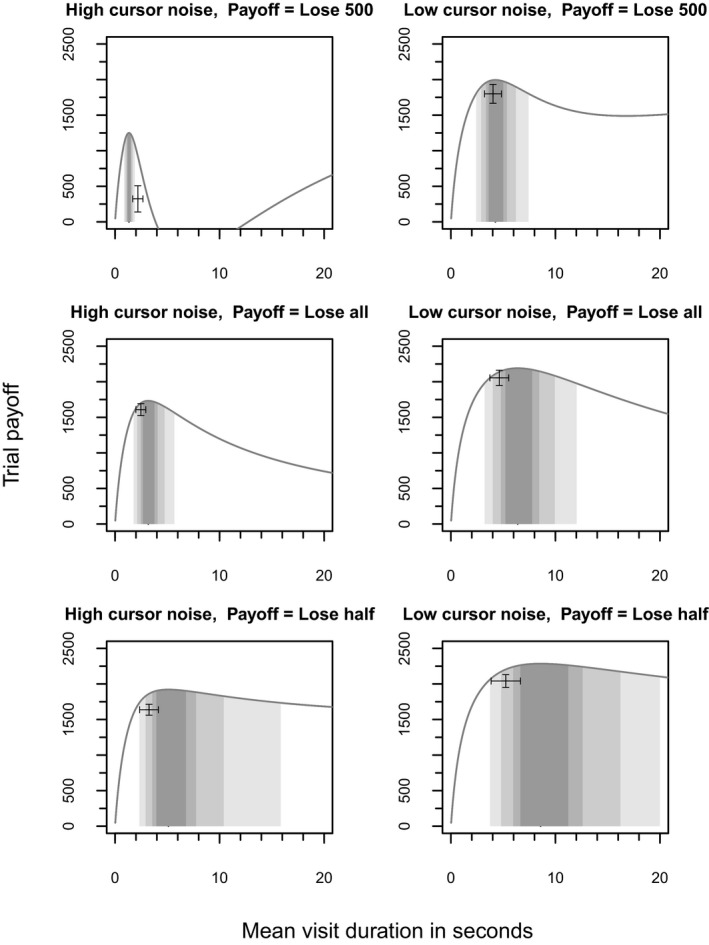
Expected trial payoff as a function of the mean typing task visit duration. The horizontal axis shows the possible visit durations participants could adopt, while the vertical axis shows the corresponding expected payoff for the trial. The payoff curves are based on the aggregate parameters across all participants. The data points show participants’ mean visit duration and achieved score with 95% confidence intervals. The darkest shaded area indicates the typing task visit durations that achieve within 1% of the maximum; the lighter shades indicate durations that achieve within 2%, 5%, and 10%, respectively.

Fig. 4 shows the shape of the modeled payoff functions for each of the conditions. The highest point on these curves represents the maximum score and therefore corresponds to the “optimal strategy.” The gray bands reveal the efficiency of participants’ chosen strategies, with darker areas highlighting visit times that achieved higher scores (with intervals within 1%, 2%, 5%, and 10% of the optimum). Only in the lose 500 high noise condition did participants choose a strategy that was not within at least 5% of the optimal score.

Fig. 4 also reveals that some conditions were harder than others. The band of strategies that achieved a score within 1% of the maximum (represented by the darkest shading) was considerably larger, and therefore easier to achieve (had strategies been selected at random), in some conditions than others. In the lose‐half low noise condition, the band of strategies achieving within 2% of the maximum covers 6.65 s, whereas in the lose 500 high noise condition, the same band covers just 0.44 s.

Qualitatively, the variance in human strategy selection corresponds to the size of the optimal payoff region for each condition. In the lose‐all high noise condition, there is a narrow region of peak reward. Correspondingly, participants show low variance in their choice of strategy. In contrast, in the lose‐all low noise condition, there is a much wider region of peak reward predicted by the model, and participants respond by showing greater variance in their choice of strategy.

In most of the conditions, participants could have spent more time typing per visit to achieve a higher payoff (i.e., the human data are often just to the left of the peak of each payoff curve in Fig. 4). In each case, the strategy adopted by participants was shorter than the optimal strategy, except for in the lose 500 high noise condition, where the chosen strategy was slightly longer than the optimal. This result suggests that our participants were being risk averse. The largest discrepancy was in the lose‐half low noise condition, in which participants chose to spend 3.33 s fewer per visit than the optimal strategy, but they were still predicted to achieve 96% of the maximum payoff.

Examining the shape of the payoff curve, we see that the lose 500 condition was unusual in that it had two points of high reward. The global maxima is a strategy that types for the whole duration of the trial (i.e., outside of the range of Fig. 4). Participants instead appeared to aim for the first *local* maximum (visible in Fig. 4), despite it being less valuable than the later global maximum. In the lose 500 low noise condition, participants spent, on average, 0.23 s fewer than required for the local maximum. However, in the high noise, lose 500 condition, participants spent an average of 0.85 s longer than required for the local maximum.

### 2.5 Experiment 1 discussion

The results from Experiment 1 reveal that participants adapted to changes in payoff function and task difficulty (i.e., statistical analyses found significant main effects of both factors). The modeling analysis suggests that participants were not adopting *the optimal strategy* (i.e., the strategy that could have earned them the highest reward). However, their strategies were very close to the optimal strategies in each condition (see Fig. 4) and were predicted to achieve on average a score that was within 94% of the maximum possible score (mean of the median efficiency across all conditions). Whether or not this performance is the limit of people's ability to optimize is difficult to assess because there were only two trials in the experiment. It is possible that prolonged experience in the environment might have allowed participants to learn more about the task environment and payoff functions, and consequently perform better.

Participants’ performance may have been further limited by transferring strategies from one condition to another in the within‐subjects design, despite that the overall optimum strategy might have changed. This strategy transfer seems likely given that participants were required to maximize their score as soon as they encountered a new payoff function and were not given time to explore alternative strategies. This type of strategy transfer has also been observed in Janssen and Brumby (2015).

## 3 Experiment 2

We designed a second experiment in order to address the above issues and provide a more in‐depth understanding of people's ability to perform optimally. We increased the number of trials from 2 to 50 and divided them into three phases. An *exploration phase* allowed participants to experiment with different strategies at no cost to them. An *exploitation phase* followed, in which we sought to test whether people could consistently maintain a near‐optimal performance. The first two phases were used to test two different payoff functions between subjects. This design allowed us to avoid any transfer of strategy from one payoff function to another.

In order to explicitly test the possibility of strategy transfer, we added a final stage in which both groups abruptly experienced a new payoff function in order to determine whether the preceding payoff function influenced the strategy they adopted.

**Figure 5:**
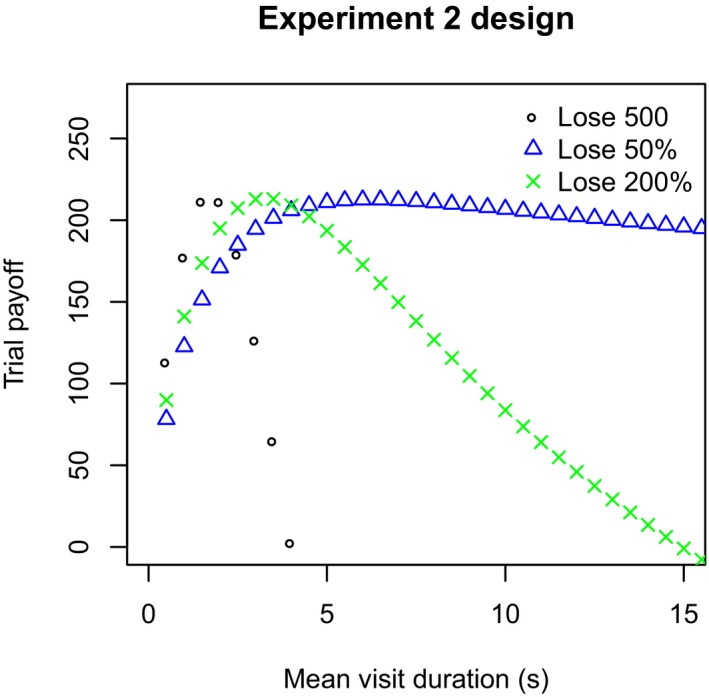
Experiment 2 expected payoff in each condition. These curves are derived from the group‐level parameters observed in Experiment 1. The “x” points show the payoff of the transfer condition, which participants experienced immediately after one of the other two payoff functions.

To have a strong test, we needed three different payoff functions that had distinct optima. We used the model and the aggregate parameters from the participants in Experiment 1 to design such a new payoff environment. As shown in Fig. 5, we settled on an environment in which the cursor's movement had a standard deviation of 4, and the target area had a radius of 120 pixels. The gain function changed such that typing a digit correctly earned four points, while a mistyped digit cost one point. In one payoff function, the penalty for allowing the cursor to exit was 500 points, and in the other, it was 50% of the previously obtained points. We devised a third payoff function in which the penalty was to lose 200% of the previously obtained points. This third payoff function was used to test for strategy transfer from the other payoff functions (see Fig. 5). Critically, all these payoff functions differed only in the nature of penalty applied, whereas environment (cursor speed and target radius) remained the same. Each of these payoff functions had different optimal strategies such that participants would need to adopt a different strategy in each to maximize their reward.

### 3.1 Methods

#### 3.1.1 Participants

Thirty participants (six male) recruited from the University College London psychology participant pool took part in the study. Each participant received £7.50 as compensation.

#### 3.1.2 Materials

The materials and tasks were identical to those in Experiment 1, with the following exceptions. In the typing task, participants earned four points per correct key press and lost one point per incorrect key press. In the tracking task, the cursor movement had a standard deviation of 4 pixels. In the dual‐task conditions, trials lasted for 30 s.

#### 3.1.3 Design

Between subjects, we tested two levels of payoff function (lose 500, lose‐half). Participants were initially allowed to explore the payoff functions and different strategies for 20 trials (exploration phase), and then given a further 20 trials to maximize their score (exploitation phase). After trying to maximize their score, both of the between‐subjects groups then experienced a new payoff function (lose 200%), called the transfer stage. These three stages were designed to allow us to measure how quickly participants learned to adapt to the payoff functions, whether their performance then remained stable, and finally whether the learned strategy would be transferred to a novel condition.

For all of the payoff functions, the task difficulty remained the same with the cursor having a standard deviation of 4 pixels on its movement. In Experiment 2, we scaled the payoff functions such that the expected amount they could earn was approximately the same in each condition, which allowed for a simpler comparison of performance between conditions. As in Experiment 1, we measured the mean visit duration to the typing task as our principal dependent variable.

#### 3.1.4 Procedure

The experimenter explained the dual‐task setup to participants, instructing them how to use the joystick and emphasizing that they should earn as many points as possible. The experimenter also informed participants that they could gain points by typing fast and accurately, but lose points by typing inaccurately or letting the cursor move outside the target circle. Details of the payoff function, however, were not revealed to participants.

The study started off with a practice session, during which participants completed two tracking‐only trials, two typing‐only trials, and two dual‐task trials. For the practice session, each tracking‐only trial lasted 10 s, each typing‐only trial 20 s, and each dual‐task trial 30 s.

In contrast to Experiment 1, participants completed 50 dual‐task trials, divided into three phases: exploration (20 trials), exploitation (the next 20 trials), and transfer (the last 10 trials). During the exploration phase, participants were instructed that their score on these trials would not count toward their total score; participants could therefore safely explore the success of different strategies. In the exploitation phase, participants were instructed to obtain as many points as possible. The final phase began with a change in the tracking penalty from either lose 500 or lose‐half (depending on the between‐subjects group) to lose 200%. Again, participants were instructed to maximize their points total, but they had no opportunity to explore different strategies.

After every five trials of these three phases, participants were allowed to take a break and could return to the task by pressing the spacebar on the keyboard. The experiment lasted 45–60 min in total.

### 3.2 Results

#### 3.2.1 Exploration and exploitation

**Figure 6:**
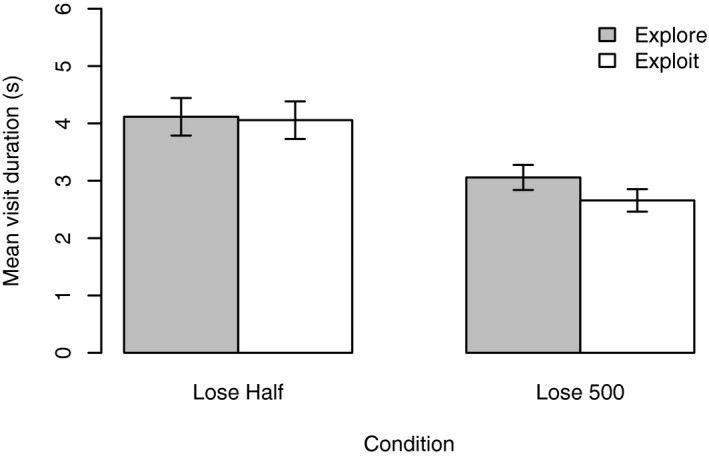
Experiment 2 results. Participants in the lose‐half condition made longer visits to the typing task than participants in the lose 500 condition during the exploitation phase. In the lose 500 condition, participants’ mean visit time decreased from the exploration to the exploitation phase. The optimal strategies were 5.89 s and 1.82 s for the lose‐half and lose 500 conditions, respectively. Error bars are standard error of the mean.

Fig. 6 shows the mean visit duration for the explore and exploit phases of the two conditions. To assess the impact of payoff condition (lose‐half, lose 500) and phase (exploration, exploitation), we conducted a 2 × 2 Mixed anova on the dependent variable of mean typing task visit duration. There was a significant main effect of phase *F*(1, 28) = 9.13, *p *=* *.005, $\eta_{p}^{2}=0.25$. Visit time was higher during the exploration phase, (*M *=* *3.59 s*,* CI* *= [3.18, 3.99]) compared to the exploitation phase (*M *=* *3.36 s*,* CI* *= [2.97, 3.75]).

There was also a significant effect of payoff function *F*(1*,* 28) = 10.42*, p *=* *.003*,* $\eta_{p}^{2}=0.27$. Visit time was higher in the lose‐half condition (*M *=* *4.09 s*,* CI* *= [3.54, 4.64]) than in the lose 500 condition (*M *=* *2.86 s*,* CI* *= [2.31, 3.41]). A significant interaction phase*payoff was also observed *F*(1*,* 28) = 5.06*, p *=* *.033*,* $\eta_{p}^{2}=0.15$. The reduction in mean visit duration was, however, larger in the lose 500 condition than in the lose‐half condition. Participants in the lose‐half condition had a mean visit duration of 4.12 s (*SD *= 1.27 s) in the exploration phase, falling slightly to 4.06 s (*SD *= 1.27 s) in the exploitation phase. Participants in the lose 500 condition had a mean visit duration of 3.06 s (*SD *= 0.85 s) in the exploration phase, falling to 2.66 s (*SD *= 0.76 s) in the exploitation phase. Similar patterns were observed in other recorded measures such as number of keys pressed (see Supporting Information).

**Figure 7:**
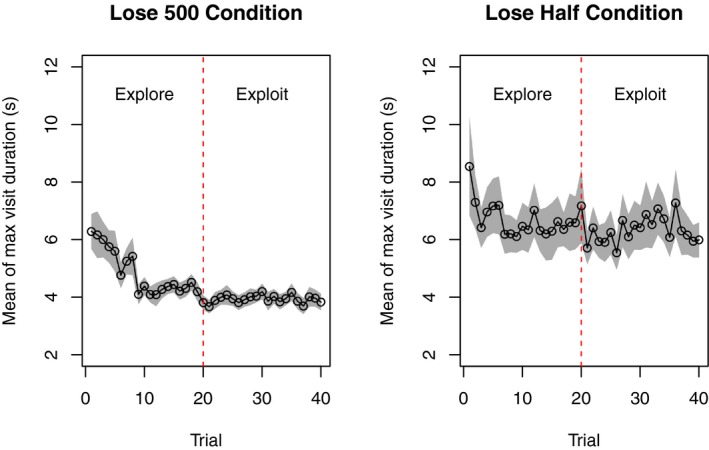
Participants’ maximum visit time during the exploration phase (to the left of the dotted vertical line) and the exploitation phase (to the right of the line). Shaded area shows standard error of the mean.

To get a sense of how the time spent on the typing task changes with task experience, Fig. 7 shows the maximum visit duration for each trial in the lose 500 condition (left) and the lose‐half condition (right). In each condition, participants reduced the amount of time spent on the typing task as the number of trials (and experience) increased. This adaptation was slightly faster (indicated by a steeper slope) in the lose 500 condition.

#### 3.2.2 Transfer

To determine whether the previously experienced condition (lose‐half, lose 500) affected the strategies participants used in the transfer phase, we conducted an independent samples two‐way *t‐*test on the mean visit duration in the transfer phase. There was a significant difference in visit duration between the groups *t*(28) = 2.93*, p *=* *.007. When the tracking penalty changed to lose 200%, participants in the lose‐half condition reduced the duration of their visits to the typing task, thereby adopting a different strategy (*M *=* *3.43 s, *SD *= 0.68). Participants in the lose 500 condition did not adjust their behavior and continued with approximately the same strategy (*M *=* *2.62 s, *SD *= 0.83).

#### 3.2.3 Model results

We modeled the results from Experiment 2 using the same model as Experiment 1 with adjustments to reflect the different payoff structure used in the second experiment. In order to examine how close to optimal the participants performed, for each participant, we ran the model using individual parameters in the exploitation phase (typing speed, error rate, and tracking time). We determined the entire strategy space available to each participant by systematically exploring the effect of different visit durations to the typing task. For each strategy, we determined how many points would have been earned on average. This analysis allowed us to identify for each participant the optimal visit duration.

Table 2 shows that there was a significant difference between participants’ scores and those of the optimal strategies. In the lose‐half condition, and in the lose 500 condition, participants’ scores were significantly lower than those predicted by the optimal strategy.

**Table 2:** Comparison of participants' mean scores and those of their optimal strategies in Experiment 2

Payoff	Participants' Score (*SD*)	Optimal Score (*SD*)	*t*(14)	*p*
Lose‐half	253 (64)	268 (60)	−2.17	.048
Lose 500	88 (164)	182 (41)	−2.32	.036

**Figure 8:**
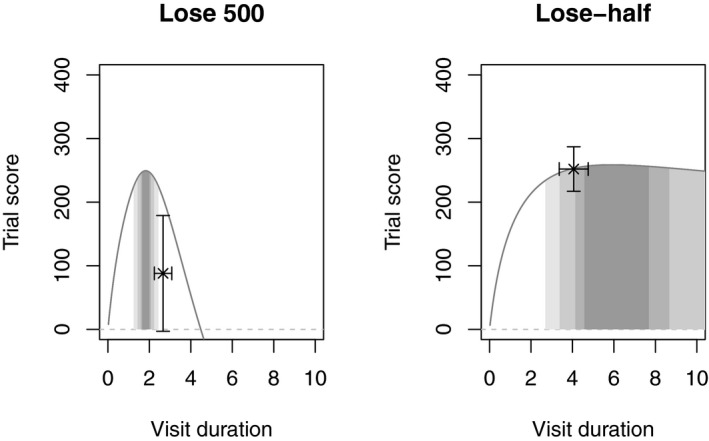
Expected trial payoff as a function of the mean typing task visit duration. The horizontal axis shows the possible visit durations participants could adopt, while the vertical axis shows the corresponding expected payoff for the trial. The payoff curves are based on the aggregate parameters across all participants. The data points show participants’ mean visit duration and achieved score with 95% confidence intervals. The darkest shaded area indicates the typing task visit durations that achieve within 1% of the maximum; the lighter shades indicate durations that achieve within 2%, 5%, and 10%, respectively.

We now examine how the shape of the payoff functions in each condition affected participants’ performance. Fig. 8 shows the shape of the expected payoff as a function of all the possible strategies in each condition. The lose 500 condition has relatively few strategies that are optimal (i.e., the dark shaded area is small), whereas the lose‐half condition is more forgiving with a large range of near‐optimal strategies. This difference is reflected in the variance of the participants’ performance: the standard error of participants’ strategies was larger in the lose‐half condition than in the lose 500 condition. Although each participant's payoff curve was slightly different depending on their individual parameters, all curves followed the basic shape seen in Fig. 8. Participants in the lose 500 condition adopted a mean visit time of 2.66 s, outside the range of optimal strategies shown in Fig. 8. Participants in the lose‐half condition adopted a mean visit time of 4.06 s—a strategy that achieves 97% of the optimum.

**Figure 9:**
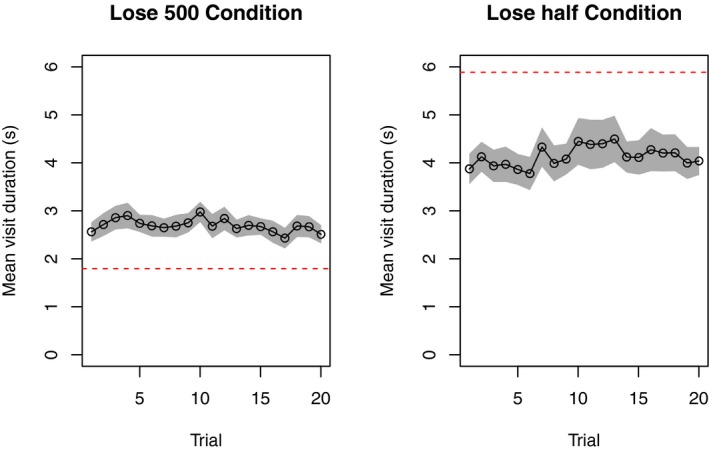
Participants’ mean visit durations during the exploitation phase of the experiment. Each data point shows the mean visit duration for that trial number. Participants maintained a consistent level of performance throughout the exploitation phase. Note that strategies were markedly different between the two payoff conditions. The dashed horizontal line shows the optimal modeled strategy for the mean participant. Shaded area denotes standard error of the mean.

Participants’ performance was not optimal in either condition, but it was stable. Fig. 9 plots the mean visit duration to the typing task for each trial of the exploitation phase; the gray shaded areas represent the standard error on the visit duration. These data show that participants adopted different strategies in the different payoff conditions, and that those strategies remained stable throughout the exploitation phase. There was no significant difference between the first and last trial visit durations in the lose 500 condition, *t*(14) = 0.38, *p *=* *0.71, or in the lose‐half condition *t*(14) = −0.79, *p *=* *.45. Fig. 9 shows that participants needed to spend less time typing in the lose 500 condition and more in lose‐half condition. The optimum strategy (shown by the dashed red line) required many more digits to be typed per visit in the lose‐half condition than in the lose 500 condition.

**Figure 10:**
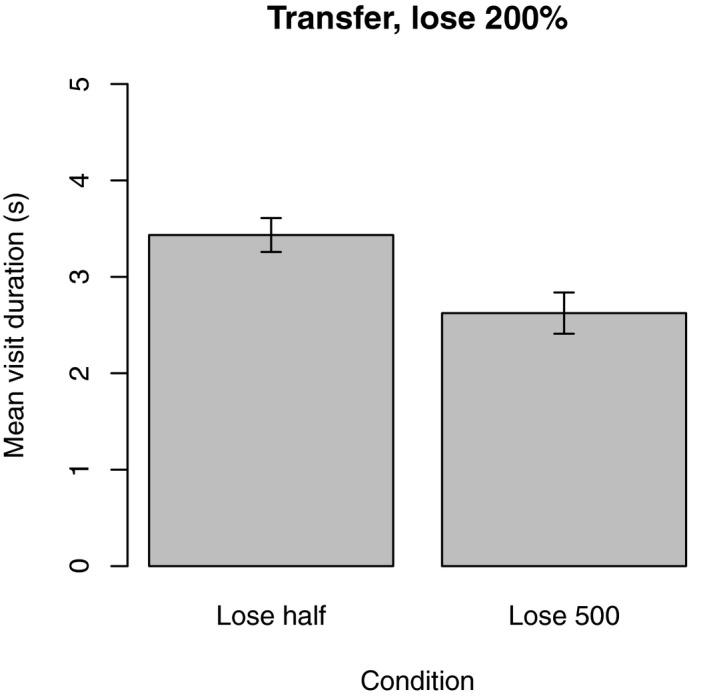
Transfer stage. Participants who experienced the transfer stage after the lose‐half condition made longer visits than those who had previously experienced the lose 500 condition. Error bars show standard error of the mean.

In the transfer stage of the experiment, participants in the lose‐half condition adjusted their strategy so that they spent less time per visit. By contrast, participants in the lose 500 condition did not significantly increase their visit duration. Fig. 10 shows that participants adopted different strategies in the lose 200% phase depending on which payoff function they had experienced immediately before.

## 4 General discussion

Overall, the results of both experiments reveal that people are able to change their strategy in response to changes in the environment. Although this adaptation falls short of being optimal, people do select strategies that are close to the optimum.

The adaptation we have seen to different payoff functions suggests that this paradigm can be used to both encourage people to change strategies when required, and to investigate the extent and limitations of our ability to perform optimally. More work is required to understand what governs the relationship between the payoff function in an environment and how quickly people settle on a strategy. Our data suggest that there may be a sweet spot in the trade‐off between task difficulty and how informative the feedback is. A very difficult task is intrinsically hard to optimize; however, a very easy task may not afford sufficiently variable feedback to enable people to change their strategy.

The results from Experiment 1 show that participants were able to strategically adjust how long they spent on one task before switching to the other. Changes in the payoff structure and task difficulty led participants to adjust their visit times to the typing task in the direction predicted by an optimal model of performance. Participants’ chosen strategies were significantly different from the exact optimal strategy, and in five of the six conditions, participants adopted a strategy that reduced the risk of a penalty at the expense of overall gain. They were nonetheless efficient, achieving the large majority of the reward available.

These results have important theoretical implications for the understanding of human multitasking. They suggest that people are flexible and adapt behavior to task priorities (in our case: to earn points). This flexibility is in contrast to claims by Salvucci and Taatgen (2008), who explain human multitasking behavior as being emergent from task structures and largely lacking in strategic (task priority) control. However, the data reported here support the notion that people are flexible in how they interleave tasks and that they alter their strategy in response to a clearly defined reward metric (Howes et al., 2009; Janssen, 2012; Janssen & Brumby, 2015; Janssen et al., 2011). This notion of flexible strategies has recently also been proposed for threaded cognition models (Nijboer, Borst, van Rijn, & Taatgen, 2016), although in that study it is proposed as a hallmark of novice behavior. In contrast, in our work, flexible interleaving in response to relative value of tasks is a hallmark of skilled adaptive behavior (similar to models in Howes et al., 2009; Janssen et al., 2011; Janssen & Brumby, 2015; Zhang & Hornof, 2014).

The results from Experiment 2 provide further evidence of the robustness of this finding. Participants again used different strategies depending on the nature of the payoff function. These strategies were also not optimal but did achieve 94% of the maximum points available in the lose‐half condition and 48% in the lose 500 condition—up from 82% and 26%, respectively, for the most similar conditions in Experiment 1. Unlike in Experiment 1, in Experiment 2, the task difficulty was kept constant and only the payoff function was varied. Furthermore, Experiment 2 had many more trials allowing us to determine how quickly participants settled on their strategy and whether their performance was stable. Participants adopted significantly different strategies in the two conditions, indicating an ability to choose a strategy in response to a payoff function. However, despite the longer exposure and the opportunity to explore, participants still chose strategies significantly worse than optimal.

Experiment 2 further suggests that participants may transfer strategies from one payoff environment to another. Participants who had previously experienced a payoff function with a more severe penalty were more cautious about changing their strategy in the face of a new payoff function presented during the transfer phase.

While our participants’ choice of strategy was predicted by a model of the optimal strategy, it is also the case that our participants were somewhat risk averse. For instance, the data from Experiment 1 show that our participants could have afforded a greater probability of incurring a penalty in five out of the six conditions (for a similar finding see Juni, Gureckis, & Maloney, 2011).

### 4.1 Implications

The way in which people interleave tasks depends on a variety of factors. The nature of the incentive, the characteristics of the task itself, and the cognitive constraints on the individual will all play a role (Howes et al., 2009; Janssen et al., 2011; Lewis et al., 2014). An important theoretical implication from our findings is that people's ability to exhibit control over their strategy selection needs to be accounted for in models of human multitasking. People do not necessarily adopt a fixed strategy but actively engage with feedback in deciding how to interleave.

Although participants adapted their behavior to the payoff functions, they did not always apply the overall most efficient strategies. It is therefore critical that future research examines the nature of the payoff functions with which participants are presented. As our results show, some payoff functions are easier to adapt to than others, and a strategy from one payoff function may be transferred to another, at least while a person adapts to a new environment. All of these factors require careful experimental design and consideration of the nature of feedback given to participants in experiments.

Our findings suggest that risk aversion may lead people to take longer to settle on a new optimal strategy when a payoff environment becomes more forgiving. By contrast, a payoff environment which becomes harsher may result in a much faster change in strategy. Participants in Experiment 2 who first experienced the lose 500 condition chose shorter visits in the transfer condition than those who had previously experienced the lose‐half condition. This difference implies that people may need different levels of time and experience with a task in order to settle on an effective interleaving strategy, depending on whether they are moving from a safe to risky environment, or vice versa. The observed pattern of risk aversion also suggests that learning might proceed more quickly when moving from safer to progressively riskier environments than it would in the opposite direction.

While our experiments suggest that people are flexible in strategy selection and can optimize reward, we also found that participants transferred strategy from previous conditions to new environments. Future work should look further into how such strategy transfer can be overcome in other settings. Many real‐world work environments will have frequently shifting priorities and incentives (e.g., changes in management), so examining the underlying causes of people's tendency to stick with old strategies or adopt new ones could have strong practical importance.

All of the above implications also suggest important considerations for the design of multitasking work. People's flexibility in strategy selection might be accommodated by designing tasks in which a variety of strategies are possible. This is particularly important since the optimal strategies will differ between individuals. Task designers should also consider, where possible, giving feedback on performance in order to exploit people's ability to settle on a strategy that will maximize their efficiency.

### 4.2 Limitations

We studied the ability to interleave two tasks in a multitasking setting. As multitasking is diverse and can involve all kinds of tasks (Janssen et al., 2015), the general human ability that we investigated here should be studied further in more specific settings. Similar to other work that has focused on discretionary task interleaving (e.g., Duggan et al., 2013; Janssen et al., 2011; Payne et al., 2007), a characteristic of our task was that participants had to interleave the two tasks and could only focus on one task at a time. In Experiment 2, participants were given an explicit exploration phase. People might not always have this opportunity in real‐world settings, but learn while they are performing.

In our study, we used an explicit payoff function to express success on the task. Although explicit feedback is not available in all everyday tasks, tasks often have an associated success rate. Making the success rate explicit, via our payoff function, offered us many advantages: It captures performance in one unit, provides an objective criterion (and allows for an associated interpretation) about which tasks should be valued or prioritized, and avoids participants from having to use internal scales to compare performance on tasks. In preceding studies, we showed that even in cases where there is not an explicit payoff function, but a subjective priority, people can adapt their behavior to prioritize tasks differently to meet verbally stated instructions (e.g., Janssen & Brumby, 2010; Janssen et al., 2012).

Our model provides a simple approximation of behavior, for which most parameters were derived from empirically observed values. This level of abstraction was chosen as it proved useful before (e.g., Janssen & Brumby, 2010, 2015; Janssen et al., 2011, 2012). However, the model could be refined by incorporating, for example, production rules to govern action selection (e.g., Salvucci & Taatgen, 2008, 2011) or fine‐grained perceptual and motor actions (Zhang & Hornof, 2014). More generally, our model is at Marr's computational level, and it is focused on determining how well participants performed compared to the best strategies available. This level of analysis affords a different type of insight focused on understanding the goal of human behavior, but it does not reveal the mechanism that supports the achievement of that goal. The level of model we have used can nonetheless be useful in understanding mechanism by combining it with theories of process (Howes et al., 2009; Lewis et al., 2014).

## 5 Conclusion

The results of two experiments and computational modeling work described here show people to be highly adept at multitasking: Participants were able to make sizable and beneficial adjustments to how much time they were giving up to a task before switching back to another task, based on the downstream cumulative reward that was achieved by such decisions. While our results do show that participants often fell short of achieving optimal dual‐task performance outcomes, we did find that participants often settled on strategies that were efficient and achieved very close to the maximum reward available across a variety of conditions. Understanding the shape of the payoff curve gives insights into why participants might have fallen short of achieving optimal performance outcomes. One reason is that it can be a difficult optimization problem (i.e., there was sometimes a narrow and constrained region of peak reward on the payoff curve) and so participants were often risk averse to avoiding overshooting. The primary contribution of this work lays in the detailed empirical and computational exploration of how people adapt to explicit feedback on performance when multitasking.

## Supporting information


**Table S1.** The mean values for other measures recorded during Experiment 1. All follow the same pattern as the key dependent variable of mean visit time reported in the main text.
**Table S2.** The mean values for other measures recorded during Experiment 2. All follow the same pattern as the key dependent variable of mean visit time reported in the main text.

## Note

1 In initial model explorations, we also investigated how performance would vary based on the amount of time spent on the tracking task (Janssen et al., 2011; Janssen, 2012). We found that performance rarely fluctuated in this setting.
