# Supplementary material for: Dividing Attention Between Tasks: Testing Whether Explicit Payoff Functions Elicit Optimal Dual‐Task Performance
Source: Cogn Sci. 2017 Jun 27;42(3):820–49. doi: 10.1111/cogs.12513 (PMC5969112; doi:10.1111/cogs.12513)
Supplement: Supplementary file 1 — Table S1. The mean values for other measures recorded during Experiment 1. All follow the same pattern as the key dependent variable of mean visit time reported in the main text. Table S2. The mean values for other measures recorded during Experiment 2. All follow the same pattern as the key dependent variable of mean visit time reported in the main text. [file COGS-42-820-s001.docx]

**Supplementary Materials**

Dividing attention between tasks: Testing whether explicit payoff functions elicit optimal dual-task performance

Farmer, G.D., Janssen, C.P., Nguyen, A.T., & Brumby, D.P.

Table S1 shows the mean values for other measures recorded during Experiment 1. All follow the same pattern as the key dependent variable of mean visit time reported in the main text.

Table S1. Experiment 1 other measures

|  |  |  | Condition | |  |  |
| --- | --- | --- | --- | --- | --- | --- |
|  |  | Low noise |  |  | High noise |  |
|  | Lose 500 | Lose-all | lose-half | Lose 500 | Lose-all | lose-half |
| Mean tracking time | 1.24 | 1.32 | 1.26 | 1.37 | 1.33 | 1.41 |
| Mean keys pressed | 10.32 | 11.87 | 12.84 | 5.77 | 6.76 | 8.44 |
| Max visit duration | 7.36 | 7.24 | 8.46 | 4.10 | 4.35 | 5.42 |

Table S2 shows the mean values for other measures recorded during Experiment 2. All follow the same pattern as the key dependent variable of mean visit time reported in the main text.

Table S2. Experiment 2 other measures

|  |  |  | Condition | |  |  |
| --- | --- | --- | --- | --- | --- | --- |
|  |  | Lose 500 |  |  | Lose-half |  |
|  | Explore | Exploit | Transfer | Explore | Exploit | Transfer |
| Mean tracking time | 1.51 | 1.40 | 1.33 | 1.22 | 1.17 | 1.17 |
| Mean keys pressed | 8.79 | 8.03 | 8.29 | 12.78 | 13.22 | 11.19 |
| Max visit duration | 7.82 | 5.29 | 4.97 | 11.29 | 8.95 | 6.89 |
